# Mapping the Various Li^+^ Jump Pathways in Li_10_GeP_2_S_12_: From Ultraslow Exchange to High-Temperature Diffusion

**DOI:** 10.1021/jacs.5c10283

**Published:** 2025-10-14

**Authors:** Annika Marko, Katharina Hogrefe, Lukas Schweiger, Florian Stainer, Jana Königsreiter, Jonas Spychala, Jakob Schwaiger, Paul Heitjans, Bernhard Gadermaier, H. Martin R. Wilkening

**Affiliations:** † Institute of Chemistry and Technology of Materials (NAWI Graz), Graz University of Technology, Stremayrgasse 9, 8010 Graz, Austria; ‡ Institute of Physical Chemistry and Electrochemistry, 26555 Leibniz Universität Hannover, Callinstraße 3-3a, 30167 Hannover, Germany

## Abstract

The solid electrolyte Li_10_GeP_2_S_12_ is known to exhibit exceptionally fast Li^+^ diffusion within its tetragonal crystal structure. Experimentally, however, the various Li^+^ hopping processes remain incompletely understood. In this study, we employed ^7^Li spin-alignment echo (SAE) NMR and complementary NMR techniques to resolve lithium-ion dynamics in LGPS over nearly 12 orders of magnitude in jump rates, from ultraslow local exchange at 90 K to fast long-range diffusion close to room temperature. Our data reveal a sequence of dynamic regimes and activation energies that reflect the dimensional evolution of Li^+^ transport, from localized 1D hopping to full 3D diffusion. The SAE NMR decay rates, which directly reflect slow Li^+^ jump rates, on the order of one jump every three seconds (0.32(2) s^–1^) at 90 K, reveal two distinct dynamic regimes with activation energies of 0.09 and 0.18 eV, suggesting a transition from 1D to 2D diffusion. At higher temperatures, a third regime emerges, characterized by an activation energy of 0.28 eV, detected by NMR relaxation and SAE NMR. This regime likely corresponds to 3D Li^+^ transport, and the measured rates are in excellent agreement with those from pulsed field gradient NMR, quasi-elastic neutron scattering, and theoretical predictions.

## Graphical abstract

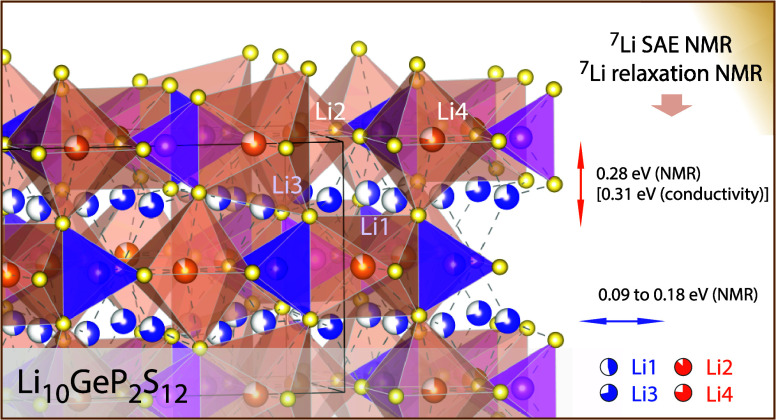

## 1 Introduction

Li_10_GeP_2_S_12_ (LGPS) is a lithium thiophosphate-based solid electrolyte that has attracted significant attention due to its exceptionally high ionic conductivity, reaching values above 10^–2^ S cm^–1^ at room temperature. First reported in 2011,^1^ LGPS belongs to a family of superionic conductors with fast Li^+^ diffusion pathways, enabled by its intriguing tetragonal crystal structure.^2,3^ Its performance rivals or even surpasses that of many liquid electrolytes, making it a promising candidate for next-generation all-solid-state lithium batteries, provided a sufficiently high electrochemical stability against lithium metal is given.^4^ So far, relatively straightforward synthesis routes have led to widespread investigations of the structural and dynamic properties of LGPS and its related compounds.^3,5–13^ In particular, nuclear magnetic resonance (NMR) spectroscopy has proven invaluable for probing lithium-ion dynamics on different time scales, offering microscopic insight into the mechanisms underpinning its fast ion transport.^14–17^ Understanding these processes is crucial for optimizing LGPS and related materials for practical energy storage applications. LGPS has also served as a test substance for methodologic nuclear magnetic spin alignment and relaxation studies.^18–20^

In 2015, Liang et al.^21^ employed ^7^Li spin-alignment echo (SAE) NMR spectroscopy to investigate Li^+^ dynamics in polycrystalline LGPS. SAE NMR is based on a principle similar to exchange NMR; however, it probes the temporal fluctuations of the electrical interactions between the quadrupolar nucleus and the nonzero electric field gradient at the nuclear site, rather than changes in local chemical shifts. Further details on the technique can be found elsewhere.^15,22–24^ In the case of LGPS, two-time SAE NMR correlation functions recorded earlier^21^ revealed a complex temperature-dependent behavior that remains not fully understood. In the present work, we extended the SAE NMR measurements down to 90 K, analyzed the decay rates by accounting for motional phase averaging effects, and complemented our study with diffusion-induced ^7^Li and ^31^P NMR spin–lattice relaxation measurements to better interpret the SAE NMR response. The jump rates extracted from NMR relaxation experiments agree very well with those reported by Ling et al., confirming that we investigated a sample with the same dynamic characteristics as in previous studies. Regarding the SAE NMR rates, we followed a different interpretation compared to Liang et al.:^21^ the increase of SAE rates at higher temperatures is attributed to a much slower diffusion process, which essentially does not contribute to the practical conductivity of LGPS. In contrast, the SAE rates influenced by motional phase averaging reflect 2D ionic transport in LGPS.

Jump rates obtained from quasi-elastic neutron scattering (QENS),^25^ pulsed field gradient (PFG) NMR,^5,26^ and theoretical investigations^3,9,12,27,28^ further contribute to building a comprehensive picture of Li^+^ dynamics in LGPS, spanning nearly 12 orders of magnitude. This comparative approach allows us to extend and contextualize previous findings, enabling a detailed and self-contained analysis of Li^+^ dynamics in LGPS.

In particular, the complex Li^+^ dynamics in LGPS are probed here using a single method, providing jump rates and activation energies in excellent agreement with previous theoretical predictions. This highlights the capability of NMR while simultaneously offering experimental validation of theoretical results. For instance, conductivity studies often report changes from one linear regime to another in Arrhenius plots because, in some cases, the methods employed cannot distinguish between different dynamic mechanisms. Our approach sheds light on the sequential nature of Li diffusion in Li_10_GeP_2_S_12_, which may also aid in interpreting both older and future data on similar LGPS-type materials.

The tetragonal crystal structure of LGPS (space group P4_2_/*nmc*), as determined by a single-crystal X-ray diffraction analysis by Kuhn et al.,^2^ is shown in Figure 1a. The framework consists of corner-sharing (Ge/P)­S_4_ tetrahedra that form a rigid polyanionic backbone, creating interstitial spaces that host mobile lithium ions. Lithium occupies several distinct sites (Li1 to Li4), coordinated by sulfur atoms to form distorted polyhedral (Figure 1a). No face-sharing Li-polyhedra are present in LGPS. Of particular interest are the Li1 (16 h, occupation factor 0.48) and Li3 (8f) sites, which form edge-sharing LiS_4_ tetrahedra aligned along the *c*-axis. These polyhedra create one-dimensional diffusion channels (Figure 1b, see also Figure S1), which are discussed to be key to the high ionic conductivity of LGPS. The connection between Li1 and Li3 sites is reported to enable fast Li^+^ hopping along these channels due to the low activation energy (see below) and relatively high site occupancy. This Li1–Li3 channel-like architecture is assumed to be central to the superior Li-ion transport in LGPS-type electrolytes. The sites Li3 and Li4 are connected by corner sharing (Figure 1c, right). The Li1–Li1 distance is only 1.63 Å (Figure 1b), giving rise to rapid, strictly localized Li^+^ jumps within the 1D channel. The Li1–Li3 jump distance is 2.36 Å (Figure 1b), which facilitates rapid Li^+^ exchange along the *c*-axis. This occurs either through direct hopping via the S–S dumbbell or along a curved pathway that takes advantage of the face-sharing tetrahedral voids connecting the distorted Li1S_4_ and Li4S_4_ polyhedra.

**Figure 1:**
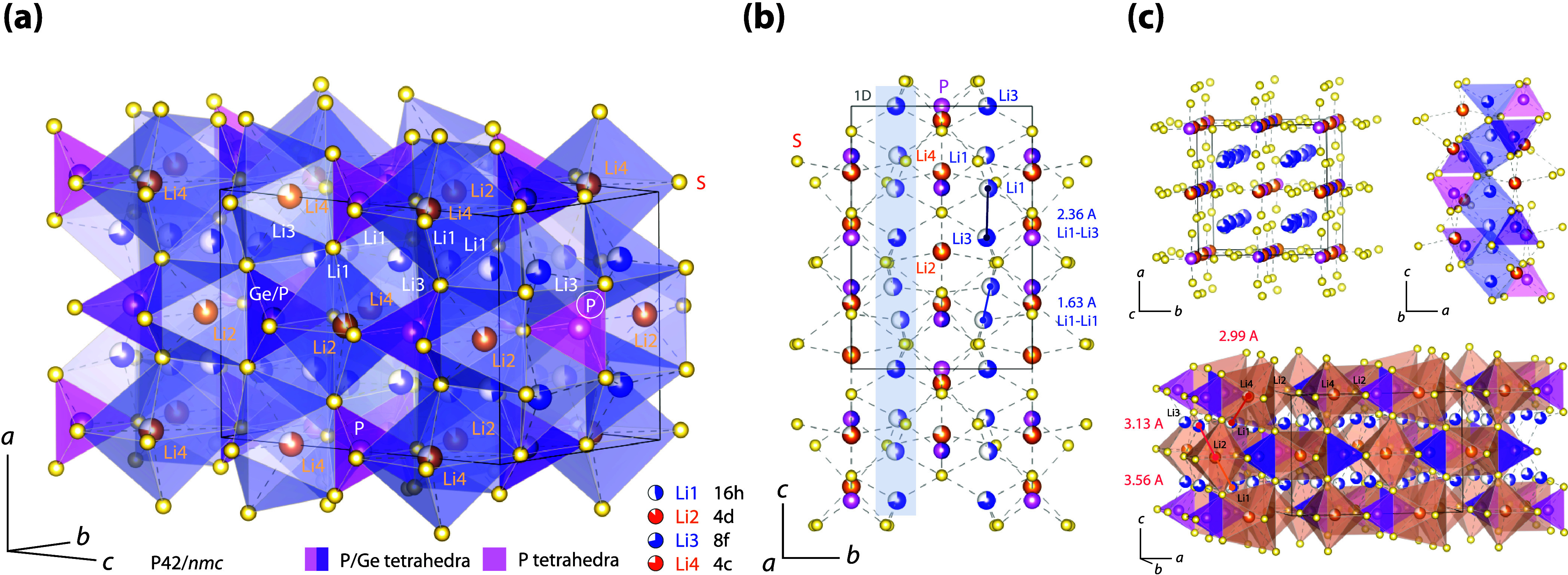
(a) Crystal structure of Li_10_GeP_2_S_12_ (*P*4_2_/*nmc*) based on the single-crystal analysis by Kuhn et al. (b) 4-fold-coordinated Li sites, Li1 and Li3, form channel-like Li^+^ diffusion pathways along the *c*-axis (see also panel c). The very short Li1–Li1 distance of 1.63 Å suggests local Li^+^ exchange within the channel. (c) Li1 and Li4 tetrahedra are connected via edge-sharing, facilitating fast Li^+^ transport. The octahedrally coordinated Li2 and Li4 sites spatially separate the 1D channels. Possible jumps from Li2 and Li4 to adjacent Li3 and Li1 sites are indicated; for example, the direct Li4–Li1 distance is only 2.99 Å, suggesting a quasi-3D diffusion network. In contrast, if Li^+^ exchange was limited to Li2 and Li4, transport would be confined to 2D diffusion within the *ab*-plane. Jumps between Li2 and Li4 would likely require intermediate positions, as their octahedra are only connected via edge-sharing. The same is expected for jumps between Li3 and Li4 with a relatively large distance of 4.4 Å, not shown.

Li2 represents an octahedrally coordinated site within the interstitial space. These sites are not part of the 1D conduction channel, but they bridge or connect neighboring channels, contributing to 3D Li-ion diffusion to some extent (see Figure 1c, see also Figure S1 showing a magnification of this illustration with further details). Bridging the Li1–Li3 channels is, as pointed out be Kuhn et al.^2^ possible via the site Li4, which connects two Li1 positions in adjacent channels with a reasonable (direct) jump distance of 2.99 Å, see Figure 1c. The distorted Li4S_6_ octahedra are connected to the Li1S_4_ tetrahdra via edge-sharing. Jumping solely within the *ab*-plane is possible when involving the sites Li4 and Li2; since the polyhedral are connected by corner-sharing, interstitial Li sites might be involved for the (2D) Li4–Li2 jump pathway.

To evaluate whether ^7^Li NMR time-domain methods can detect the diffusion pathways discussed, we employed ^7^Li SAE NMR^15,29^ in combination with ^7^Li NMR spin–lattice relaxation measurements.^14,30^ In addition, we utilized ^31^P NMR relaxation experiments, which offer more direct insight, as the spin-1/2 ^31^P nucleus senses only dipolar magnetic interactions caused by the hopping of the Li ions in its vicinity.^31^ In this way, the ^31^P nucleus acts as a “spy”, enabling indirect probing of Li-ion dynamics, a strategy previously demonstrated, for example, in our study of Li diffusion in argyrodite-type Li_6_PS_5_I.^31–33^

## 2 Experimental Section

The polycrystalline Li_10_GeP_2_S_12_ sample was prepared via conventional solid-state synthesis; details of the procedure are reported elsewhere.^13^ In this study, we used the same sample previously characterized by X-ray powder diffraction, ^31^P magic-angle spinning (MAS) NMR, as well as conductivity and electric modulus measurements.^13^ The sample was fire-sealed in glass ampules to preserve its integrity and ensure long-term protection from moisture and humidity.

^7^Li and ^31^P NMR measurements were performed on a Bruker Avance III solid-state NMR spectrometer equipped with a shimmed cryomagnet operating at a nominal magnetic field strength of 7 T. This field corresponds to a resonance frequency of ω_0_/2π = 116 MHz for ^7^Li and 121 MHz for ^31^P. For temperatures above room temperature, a commercial solid-state broadband probe (Bruker BioSpin) was used, allowing for short π/2 pulses durations of approximately 2 to 3 μs depending on the temperature. The sample chamber was flushed with cooled N_2_ gas and temperature was controlled using a Eurotherm controller with type T thermocouples. Low-temperature measurements down to 90 K were performed with a Bruker cryo probe using freshly evaporated nitrogen or helium gas. The probe, equipped with a LakeShore 331 unit and two Cernox sensors, monitored the temperature near the sample in a Duran glass ampule. Calibration with materials showing sharp phase transitions, such as Li_6_PS_5_I, ensured measurement errors remained below 2 K down to 90 K. The entire setup, which ensures highly stable gas flows and includes temperature sensors placed in the immediate vicinity of the sample, is designed to allow long-term measurements under stable temperature conditions. This situation, for static time-domain NMR, differs from those of magic-angle spinning NMR, where internal temperature calibration substances are required to, e.g., estimate temperature gradients across the rotors.

^7^Li NMR spin–lattice relaxation experiments in the laboratory frame of reference were conducted using the saturation-recovery pulse sequence. A train of ten π/2 pulses, separated by 80 μs, was applied to destroy any longitudinal (macroscopic) magnetization *M*_*z*,0_. The subsequent recovery was monitored via a detection pulse at variable delay times *t*_d_. The area under the resulting free induction decays (FID) as a function of *t*_d_ yields the magnetization transients *M*_*z*_(*t*_d_), from which the temperature-dependent spin–lattice relaxation NMR rate 1/*T*_1_ was extracted. Additional experimental details are available elsewhere.^34^ ^7^Li and ^31^P NMR rotating-frame relaxation measurements were carried out using the spin-lock pulse sequence. After a π/2 pulse turns the equilibrium magnetization *M*_*z*,0_ of the fully relaxed spin system into the (*xy*)′-plane; a subsequent pulse with a low amplitude *B*_1_ was applied to lock the transverse magnetization *M*_*xy*_.^15,34^ The decay of *M*_*xy*_ during the locking period, induced by Li^+^ hopping processes, was probed for varying locking durations *t*_lock_. The area of the corresponding FIDs plotted against *t*_lock_ yielded the relaxation transients *M*_*xy*_(*t*_lock_), from which the rotating-frame relaxation rate 1/*T*_1ρ_ was obtained. Full longitudinal recovery of *M*_*z*_ was ensured between successive experiments. The time dependence of the ^7^Li NMR magnetization transients in the laboratory frame was analyzed by parametrizing the curves with stretched exponential functions. Stretching exponents ranged from 0.7 to 1. Such shapes could point to a small distribution of relaxation rates. Dimensionality effects and the quadrupolar nature of the ^7^Li nucleus might easily produce nonexponential transients. The corresponding transients in the rotating frame of reference showed indeed biexponential time behavior, see Figure S2 for further details.

Variable-temperature ^7^Li NMR line shapes were recorded using single-pulse (π/2) excitation. Up to 64 scans were accumulated per spectrum, which was obtained directly by Fourier transformation of the corresponding zero-filled FIDs.

Variable-temperature ^7^Li NMR sinus–sinus^35^ SAE decay curves *S*_2_ were recorded using the same spectrometer employed for the 1/*T*_1_ measurements. To generate stimulated, or spin-alignment, echoes,^15^ we used the Jeener-Broekaert three-pulse sequence to create quadrupolar order: (π/2)_*y*_ – *t*_p_ – (π/4)_*x*_ – *t*_m_ – (π/4)_ϕ_ – *t*_p_ – echo (acquisition).^23,35–39^ Decay curves were acquired at fixed preparation times *t*_p_ and varying mixing times *t*_m_; in some experiments, *t*_p_ was also varied between 10 and 210 μs. Up to 18 different mixing times were used to construct each decay curve *S*_2_(*t*_p_ = 10 μs, *t*_m_), with up to 128 signals accumulated per echo. A 32-step phase cycle was applied to suppress unwanted coherences, and a recycle delay of 5*T*_1_ was maintained between each scan. Further details can be found elsewhere.^35^ Depending on the temperature, the mixing time ranged from 10 μs to 100 s. The echo decay curves, or two-time correlation functions, were analyzed with either a stretched single exponential or with a sum of two exponentials to extract the individual decay rates governing echo damping. At low temperatures, *S*_2_ reflects the loss of phase coherence due to Li^+^ jump processes (1/τ_SAE_). Motional phase averaging and quadrupolar spin–lattice relaxation effects contribute to the echo decay at higher temperatures. The curves are completely reproducible and do not depend on the heating or cooling protocol. Similarly, the ^7^Li and ^31^P NMR relaxation rates, as well as the ^7^Li NMR spectra measured during multiple heating and cooling cycles up to around 500 K, show no dependence on the temperature path.

## 3 Results and Discussion

^7^Li SAE NMR can be used to probe slow Li^+^ exchange processes, provided the ions (spin-3/2 nuclei) hop between sites with inequivalent quadrupolar parameters within the crystal structure. The upper limit of detectable jump rates is roughly determined by the spin–spin relaxation rate, allowing the detection of rates up to approximately 10^4^ s^–^^1^. The lower limit is set by the quadrupolar spin–lattice relaxation rate 1/*T*_1Q_, typically on the order of 1/*T*_1_. To access such slow Li^+^ dynamics, we cooled the LGPS sample to temperatures as low as 90 K. Figure 2a displays the two-time *S*_2_(*t*_p_ = 10 μs, *t*_m_) correlation functions measured at a fixed preparation time *t*_p_, with varying mixing times. Dashed and solid lines represent fits using stretched exponentials *S*_2_ ∼ exp­(−(*t*_m_/τ_SAE_)^γ^); in the ideal case the decay rate 1/τ_SAE_ corresponds to the Li^+^ jump rate 1/τ. The stretching factors γ are indicated and range from 0.70 (90 K) to 0.24 (159 K). The corresponding echo decay rates are shown in the Arrhenius plot of Figure 2b.

**Figure 2:**
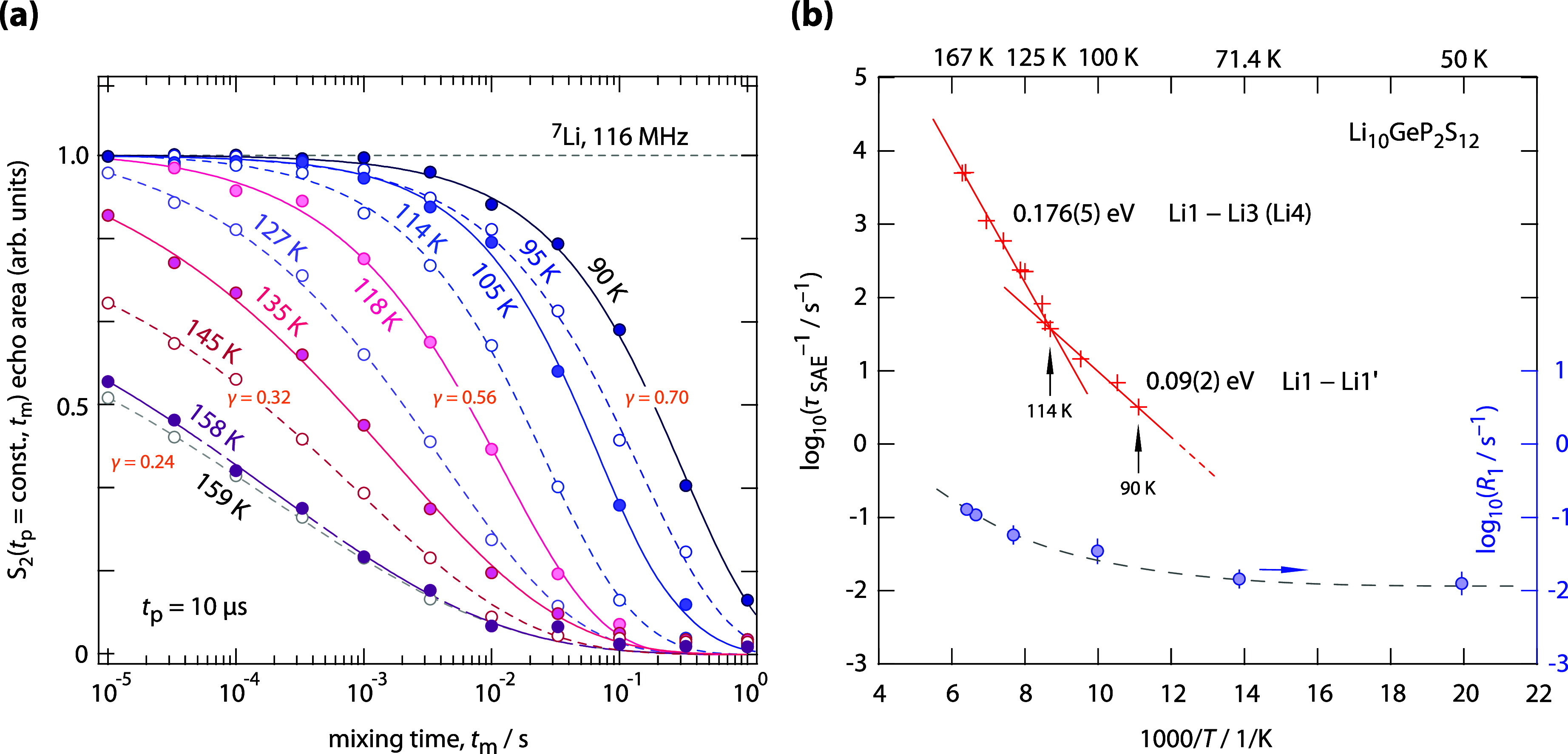
(a) *S*_2_ (sin-sin) two-time single-spin correlation functions obtained from ^7^Li SAE NMR (116 MHz, *t*_p_ = 10 μs) on polycrystalline Li_10_GeP_2_S_12_. The amplitudes of the curves were normalized to range between 0 and 1. Dashed and solid lines represent fits using stretched exponentials; γ denotes the stretching exponent. (b) Arrhenius plot showing the temperature dependence of the decay rates 1/τ_SAE_(1/*T*), along with independently measured spin–lattice relaxation rates 1/*T*_1_ (right axis), which define the lower limit of detectable Li^+^ jump rates. An activation energy of 0.09 eV characterizes regime I, whereas regime II exhibits an activation energy of 0.176 eV.

It is evident that 1/τ_SAE_(1/*T*) exhibits two distinct dynamic regimes, characterized by activation energies of 0.09(2) eV (regime I) and 0.176(5) eV (regime II). Activation energies as low as 0.09 eV, here also seen in 1/*T*_1_ NMR relaxation (see below), have been predicted for Li^+^ translational jumps within the channel-like arrangement formed by the Li1 and Li3 sites.^3^ Here, we attribute the low-temperature regime, associated with this smaller activation energy, to spatially restricted Li^+^ exchange processes occurring within the Li1–Li1 configuration where the Li^+^ ions are separated by only 1.63 Å. For this kind of jump processes, values as low as 0.03 eV have been reported.^3^ Even for Li^+^ ions hopping between crystallographically very similar sites, the temporal fluctuations of the associated quadrupole frequencies are expected to induce echo damping. Jumps between Li1 and Li3 can also contribute to this hopping process, as suggested by theory (vide infra). At higher temperatures, averaging effects might be expected, as will be discussed below.

The regime II observed above 120 K likely reflects jumps between Li1 and Li3 sites within the channel-like framework of LGPS. As compared to regime I, the likelihood that exchange processes involving the Li4 (and possibly Li2) sites also contribute to the echo decay in this temperature range is certainly higher. The fact that multiple sites contribute to the echo decay is supported by the stronger variation of the stretching exponent γ in regime II (from 0.56 to 0.24; see Figure 2b), indicating increased dynamic heterogeneity at higher temperatures. In contrast, spatially restricted Li1–Li1 (and Li1–Li–Li3) jumps produce a comparatively weak temperature dependence of γ in regime I (from 0.70 to ∼0.6). In summary, we attribute the activation energy of 0.09 eV to localized, intrachannel (1D) Li^+^ diffusion. The higher value of 0.176(5) eV is associated primarily with Li^+^ exchange between Li1 and Li3 sites, which enables long-range 1D diffusion along the *c*-axis, but we cannot rule out the involvement of processes associated with the Li4 site.

With increasing temperature, specifically from 159 to 178 K, a pronounced change in the S_2_ decay curves is observed. First, the center of the decay shifts toward longer mixing times, resulting in lower decay rates than would typically be expected at higher temperatures. Second, the S_2_ echo amplitude no longer decays fully to zero but instead levels off at a plateau value of *S*_2_ ≈ 0.23 at *t*_m_ = 0.1 s. The final decay to *S*_2_ = 0 at sufficiently long mixing times occurs at a rate that matches the spin–lattice relaxation rate, 1/*T*_1_. These two features indicate that rapid Li^+^ exchange between the involved electrically inequivalent sites leads to partial averaging of the quadrupole frequencies, thereby reducing the efficiency of echo damping.^29^ As a result, the decay curves shift toward longer mixing times, and the stretching exponent γ, if interpreted as a measure of dynamic heterogeneity or distribution of decay rates, increases due to motion-induced frequency averaging. Heavily stretched curves are expected when the distribution of decay rates is broad.

The emergence of a plateau *S*_∞_ in the *S*_2_ curves, reaching amplitudes as high as 0.4 (Figure 3a), indicates a limited number of electrically inequivalent Li^+^ positions. This reduction is a direct consequence of motion-induced averaging of the electric quadrupolar interactions.^29^ Li spins belonging to a subset of ions that rapidly exchange between crystallographically distinct sites experience an averaged electric field gradient. For this subset, the echo amplitude decays only due to conventional quadrupolar spin–lattice relaxation,^24^ 1/*T*_1Q_, as evidenced by the second, slower decay step. Notably, the corresponding decay rates closely match the independently measured 1/*T*_1_ values. Since 1/τ_SAE_ of the initial decay step also approaches 1/*T*_1_ (see the double arrow in Figure 3b), this similarity may complicate an accurate determination of the plateau amplitude, which decreases with increasing temperature (Figure 3a). When 1/τ_SAE_ approaches 1/*T*_1_, a separation is no longer possible; this situation near ambient temperature may also affect the determination of *S*_∞_.

**Figure 3:**
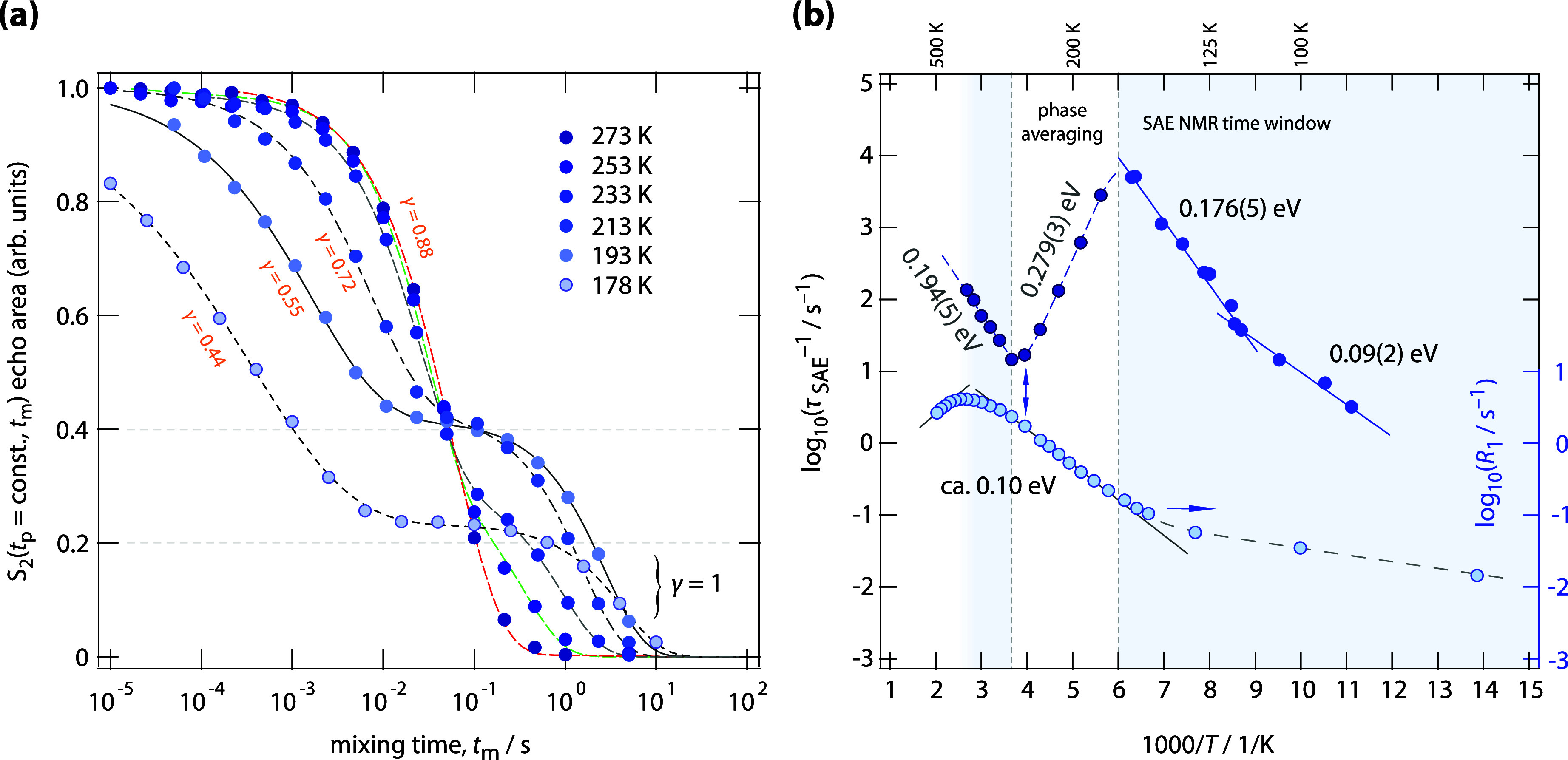
(a) ^7^Li SAE NMR two-time correlation functions of Li_10_GeP_2_S_12_ (*t*_p_ = 10 μs) recorded at the indicated temperatures. The stretching factors of the exponential functions (solid and dashed lines) used to fit the *S*_2_ curves are also included. The second decay step has been taken into account with a nonstretched exponential (γ = 1). (b) Temperature dependence of the SAE NMR decay rates, including data from *S*_2_ curves recorded at temperatures up to 373 K. For comparison, the corresponding ^7^Li NMR 1/*T*_1_ relaxation rates are shown as well. These exhibit a motion-induced peak, from which a mean Li^+^ residence time of approximately 1.4 ns at 370 K can be deduced.

^7^Li SAE NMR *S*_2_ curves recorded at the high-temperature limit of the SAE dynamic window are shown in Figure 4. The curve, measured at 168 K and *t*_p_ = 15 μs, already exhibits a shallow final-state amplitude *S*_∞_ of 0.1, which remains nearly constant with respect to the evolution time. Compared to the curve obtained at a slightly lower temperature of 159 K (see Figure 2a), the stretching factor already shows a modest increase, rising from 0.24 to 0.34 at 168 K. This observation reflects a continuous change in curve shape, decay rates, and plateau amplitude with increasing temperature.

**Figure 4:**
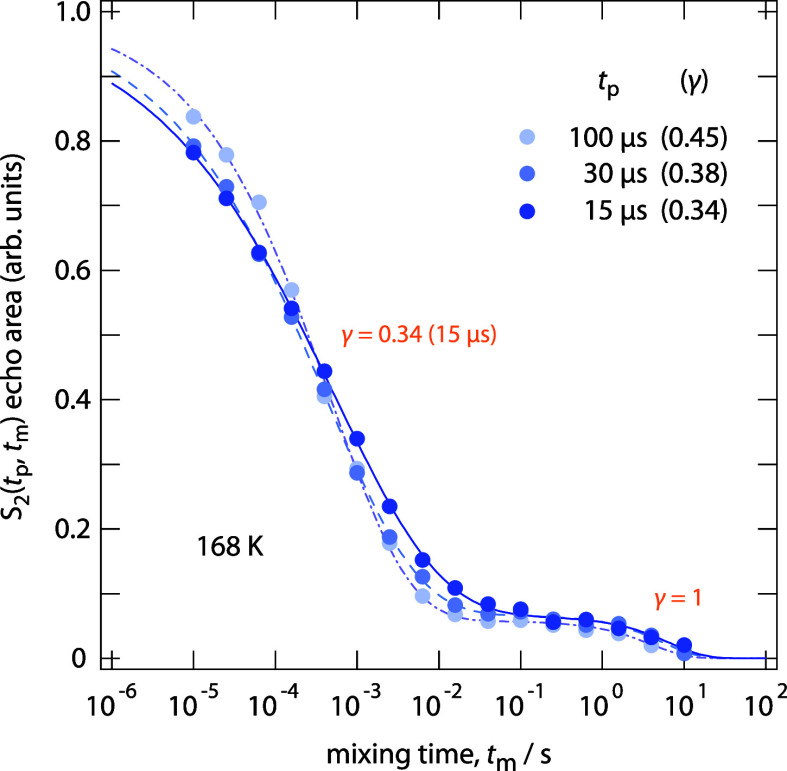
Normalized ^7^Li SAE NMR two-time correlation functions, scaled to range between 0 and 1, were recorded at 168 K, a temperature within the transition regime that connects the true SAE NMR time window with the range where motional averaging processes begin to dominate the echo decay. The curves were acquired at the indicated evolution times *t*_p_. Since the final-state amplitudes show no significant dependence on evolution time, the increase observed in Figure 3a can be mainly attributed to a temperature effect.

We attribute the activation energy of 0.279(3) eV (Figure 3b) associated with the first *S*_2_ decay step of the curves shown in Figure 3a to motion-induced averaging of quadrupolar interactions involving Li1, Li3, and Li4 sites. The rapidly growing involvement of Li4 (and possibly Li2) in this process contributes to the average activation energy of approximately 0.28 eV. A comparable value has indeed been proposed for Li^+^ diffusion within the *ab*-plane of LGPS by the very first molecular dynamics simulations on LGPS by Mo et al.^27^ In addition, Huang et al.^12^ report on 0.34 and 0.23 eV for Li^+^ hopping involving the Li2 and Li4 sites, respectively (see also below). For comparison, the bulk ionic conductivity of the LGPS sample investigated in this study exhibits an activation energy of 0.31 eV in the same temperature range.^13^ As already indicated, we cannot rule out that Li2 sites also contribute to this process. Given that a slower diffusion process is observed by SAE NMR at even higher temperatures (see Figure 3b), it is also possible that this process involves Li jumps associated with these interchannel sites.

As is clearly seen in Figure 3b, at temperatures above 293 K, 1/τ_SAE_(1/*T*) begins to increase again, the Arrhenius line is characterized by an activation energy of 0.194(5) eV. This increase, which is also seen in ^31^P spin–lattice relaxation NMR (0.18 eV, see Figure S2, Supporting Information) suggests the involvement of additional Li sites in the overall diffusion process. In general, SAE senses extremely slow exchange processes between (electrically) inequivalent Li sites; the increase of the SAE rates, as is seen here, is controlled by a new diffusion process that comes into play only at sufficiently high temperatures. As already mentioned, one possible candidate involved in this slow process is the Li2 site, which has been proposed to remain inactive during the extremely fast Li^+^ exchange observed at lower temperatures. Nevertheless, it is also highly plausible that other, typically unoccupied interstitial sites are involved. The slower exchange processes, reflected in the low values of 1/τ_SAE_ at high temperatures (Figure 3b), do not significantly contribute to the exceptionally high ionic conductivity of LGPS. The absolute SAE decay rates in this temperature range point to a diffusion process much slower than expected even for Li^+^ exchange within the *ab*-plane; this interpretation contrasts with that of Liang et al.^21^ who have seen the same increase but with a slightly larger activation energy of 0.26 eV. Instead, the key transport processes are only those active below 293 K (and especially below 167 K), as captured by SAE NMR. For comparison, the diffusion-induced 1/*T*_1_(1/*T*) ^7^Li NMR rate peak, included in Figure 3b, suggest that at 370 K the Li^+^ jump rate is in the order of the angular Larmor frequency, 1/τ ≈ 7.3 × 10^8^ s^–1^. At this temperature, SAE NMR is able to probe a very slow diffusion process characterized by rates in the order of 10^2^ s^–1^, which is negligible for overall Li^+^ hopping in LGPS.

Interestingly, only SAE NMR provides access to a range of distinct activation energies, whereas the temperature dependence of the ^7^Li NMR spin–lattice relaxation rate, 1/*T*_1_(1/*T*), reveals only a single activation energy derived from the low-temperature flank of the relaxation peak (Figure 3b). We propose that in this regime, longitudinal relaxation, indicating an activation energy of 0.1 eV, is predominantly governed by rapid dipolar and quadrupolar fluctuations associated with Li^+^ hopping *within* the channel-like framework, as this activation energy closely matches that observed by SAE NMR in regime I (0.09 eV). In general, it is important to note that activation energies extracted from the low-temperature side of the relaxation peak are mainly influenced by short-range dynamics. In the case of NMR relaxation correlation effects arising from (local) structural disorder and Coulomb interactions can impact the activation energies in this temperature regime.^40,41^ At even lower temperatures the rates 1/*T*_1_(1/*T*) enter a ‘nondiffusive’ regime, exhibiting a weaker-than-activated temperature dependence. In this range, relaxation may be dominated by lattice vibrations or by coupling of the spins to paramagnetic impurities.^22^

To investigate the successive diffusion processes in LGPS and the influence of motion-induced averaging of quadrupolar interactions, we recorded variable-temperature ^7^Li NMR line shapes. A representative series of spectra is shown in Figure 5. As expected for spin-3/2 nuclei experiencing moderate quadrupolar interactions, the spectra at low temperatures (e.g., 7 or 25 K) consist of a central transition and a broad quadrupolar “foot” (Figure 5b). The central line reflects contributions from all magnetically inequivalent Li sites in LGPS, and the same holds true for the quadrupolar component. The observed width of 73 kHz for the quadrupolar signal (Figure 5b) suggests an average quadrupolar coupling constant of approximately 146 kHz, assuming axially symmetric electric field gradients.

**Figure 5:**
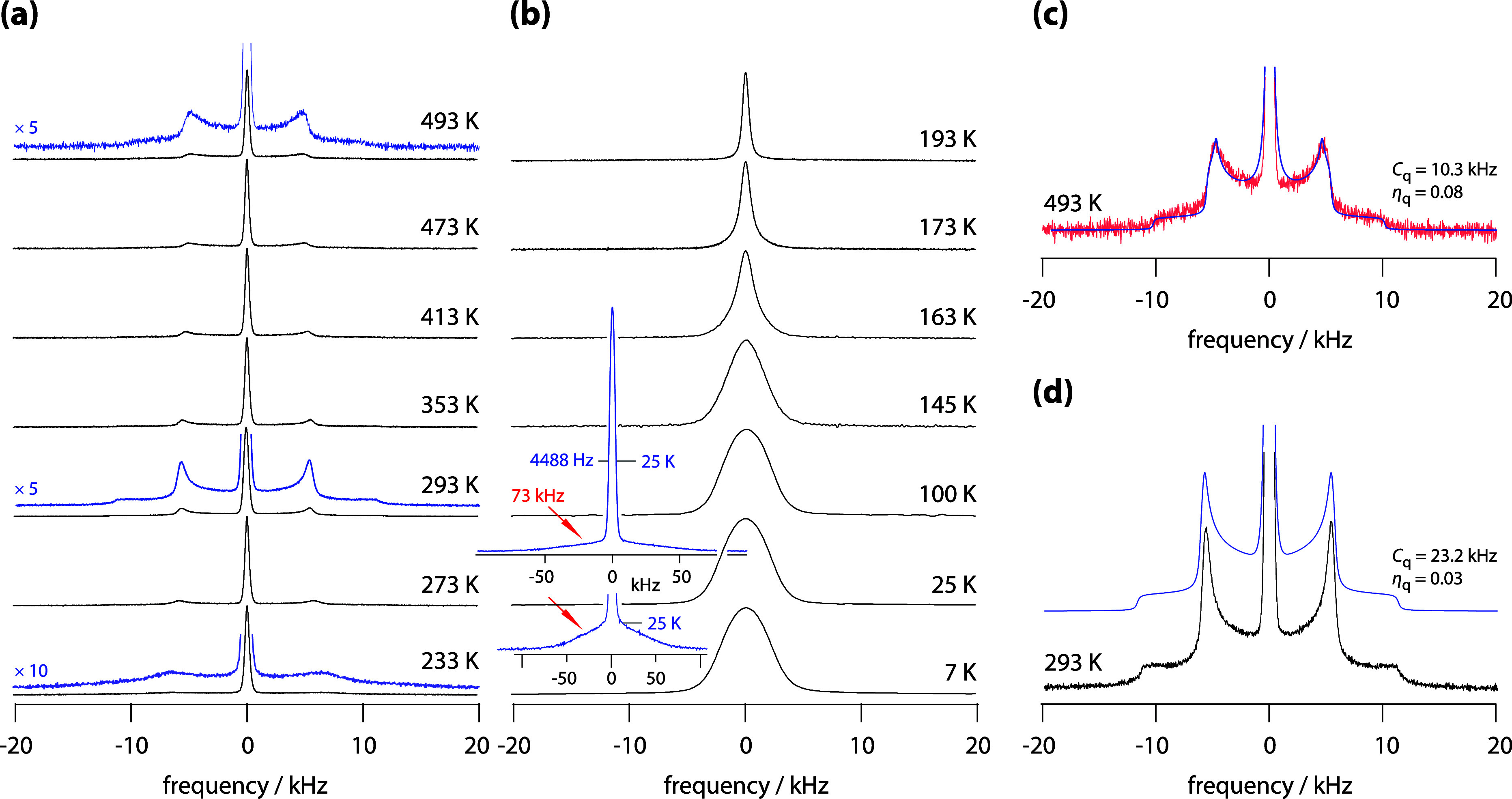
(a) and (b) ^7^Li NMR spectra of polycrystalline Li_10_GeP_2_S_12_ (116 MHz) recorded at the indicated temperatures. Insets and magnified views are included to highlight the weak quadrupolar features. The spectrum recorded at 7 K consists of a narrow central line superimposed on a much broader, featureless quadrupolar signal with an approximate width of 70 kHz. (c) and (d) Magnified views of the spectra recorded at 493 (c) and 293 K (d). The simulated patterns were generated using a single set of parameters for first-order electric quadrupolar coupling, yielding the coupling constants *C*_q_ and asymmetry parameters η_q_ as indicated. Solid lines represent the simulations, which, particularly for the spectrum acquired at 293 K, reproduce a well-resolved spin-3/2 quadrupolar pattern, indicative of an (averaged) axially symmetric electric field gradient sensed by the mobile spins.

Above approximately 145 K, we observe incipient motional narrowing of the central transition. At 163 K (Figure 5b), significant narrowing becomes evident (see Figure 6 for the full curve), coinciding precisely with the temperature at which SAE NMR decay rates start to decline. At this point, the quadrupolar foot also disappears, indicating the onset of motion-driven averaging of quadrupolar interactions. Again, this observation aligns well with the decrease in SAE decay rates (Figure 3b) in the same temperature range.

**Figure 6:**
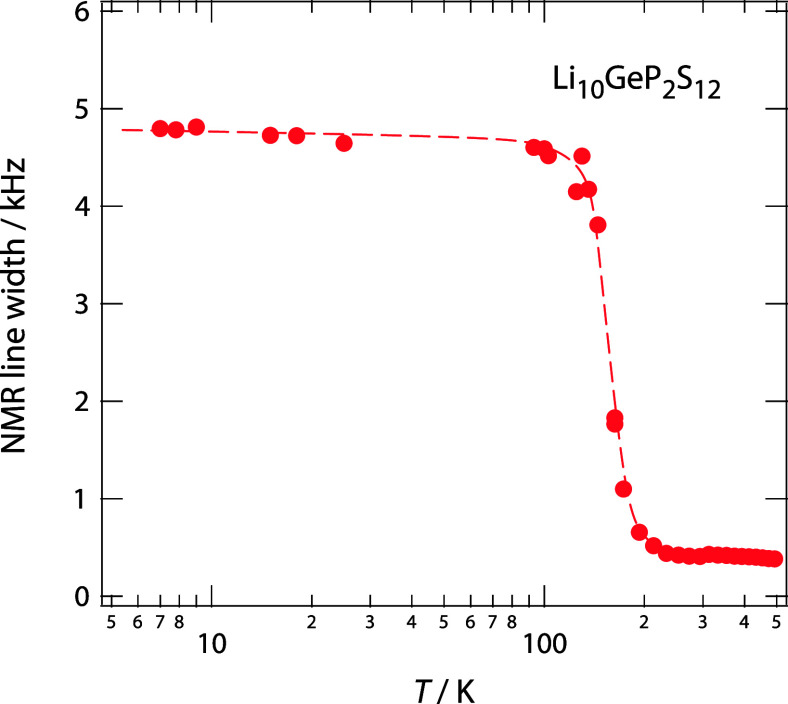
Evolution of the width of the ^7^Li NMR central line (full width at half-maximum) of Li_10_GeP_2_S_12_ (116 MHz) as a function of temperature. At temperatures as low as 7 K, the width amounts to approximately 4.8 kHz. Narrowing takes place in a rather narrow temperature range and starts at temperatures slightly larger than 100 K. Finally, an extremely narrowed line is reached already at 233 K, whose width of 387 Hz is only governed by inhomogeneties of the external magnetic field.

Slightly above 220 K, a new quadrupolar powder pattern emerges, which becomes progressively sharper with increasing temperature (see the spectra recorded at 233 and 293 K in Figure 5a). A magnified view of the spectrum at 293 K is shown in Figure 5d, clearly revealing both the 90 and 180° singularities characteristic of a spin-3/2 system interacting with an (averaged) axially symmetric electric field gradient. This pattern can be accurately simulated using a single set of quadrupolar parameters, consistent with a motionally averaged environment. The quadrupolar coupling constant *C*_q_ is found to be 23.2 kHz, with an asymmetry parameter η_q_ of 0.03. This quadrupolar interaction dominates the SAE NMR decay rates in the temperature range where the Arrhenius analysis (Figure 3b) yields an activation energy of 0.279 eV.

At even higher temperatures, the sharp quadrupolar pattern begins to broaden slightly, with this process setting in around 350 K. This temperature coincides with the onset of the second increase in SAE decay rates, which are governed by the lower activation energy of 0.194 eV. A magnified view and a simulation of the spectrum acquired at 493 K is shown in Figure 5c. The observed change in the quadrupolar pattern further supports our interpretation that another, much slower Li diffusion process becomes active on the SAE NMR time scale, as suggested above.

To compare the SAE NMR decay rates with Li^+^ jump rates obtained from other experimental techniques and theoretical calculations, we constructed an Arrhenius plot (Figure 7) that combines our low-temperature data with high-temperature results derived from ^7^Li and ^31^P (spin-lock) relaxation NMR (see Figure S1, Supporting Information) and data from PFG NMR measurements of Kuhn et al.^5^ and Kurima et al.^26^ Additionally, data from QENS of Hori et al.^25^ have been included. For this comparison, we used the Einstein–Smoluchowski equation to convert these coefficients into jump rates (see Supporting Information). In NMR relaxation, we relied exclusively on the diffusion coefficients from the maxima of the rate peaks. In general, activation energies from the low-temperature flanks of the NMR peaks may be influenced by correlation effects and local disorder.

**Figure 7:**
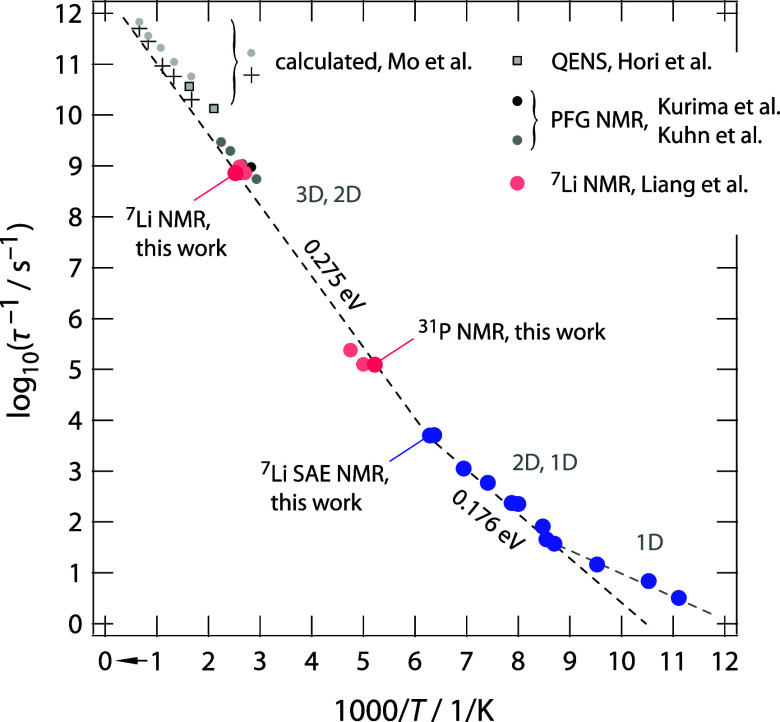
Jump rates characterizing Li-ion dynamics in polycrystalline Li_10_GeP_2_S_12_. Data from ^7^Li NMR, partly taken from Liang et al.,^21^ see key, and ^31^P NMR relaxation experiments (Figure S1) show excellent agreement with literature results obtained from PFG NMR.^5,26^ Extrapolation to higher temperatures also reveals very good consistency with jump rates derived from QENS.^25^ At low temperatures, ^7^Li SAE NMR demonstrates that the Arrhenius line describing Li-ion dynamics at ambient temperature (0.275 eV) transitions into regimes with lower activation energies. These likely correspond to intrachannel Li^+^ exchange processes. The activation energy of 0.275 eV is in perfect agreement with that obtained from SAE NMR in the regime where motion-induced averaging begins to influence echo damping. The calculated values taken from Mo et al.^27^ refer to both in-plane and overall Li^+^ diffusion. See text for further details.

Figure 7 shows that, starting from low temperatures, where ^7^Li SAE NMR can directly probe Li-ion dynamics in LGPS, the Li^+^ jump rates transition into an Arrhenius regime characterized by an activation energy of 0.275 eV. This value, which describes ion transport near ambient temperature, is again in excellent agreement with the activation energy of 0.31 eV for bulk ionic conductivity in LGPS, as recently determined by some of us through impedance spectroscopy on the same sample.^13^ It also matches the activation energy (0.279 eV) observed in the temperature range where SAE NMR is influenced by motion-induced averaging effects, as discussed above (Figure 3b, see the regime up to 250 K).

Importantly, the jump rates obtained from ^7^Li and ^31^P NMR relaxation experiments (Figure S1) not only agree with the NMR relaxation data reported by Liang et al.,^21^ but also with rates referring to diffusion coefficients from PFG NMR^5,26^ and jump rates from QENS (Figure 5).^25^ While PFG NMR probes macroscopic diffusion parameters, QENS, as a complementary tool, is sensitive to much faster Li^+^ hopping processes on the ps time scale, and thus also on shorter length scales.

For the data determined from calculations and also included in Figure 7, we refer to the early study of Mo et al., who investigated Li^+^ transport in LGPS by ab initio molecular dynamics simulations.^27^ While the upper data points (dots in light gray in Figure 7) originally refer to Li^+^ diffusion coefficients along the *c*-direction (0.17 eV); the lower values (crosses) to diffusion coefficients for Li^+^ motions within the *ab*-plane (0.28 eV).^27^ Again, we used the Einstein–Smoluchowski equation to translate these coefficients into jump rates (see Supporting Information, which also contains a comment on the influence of possible correlation factors.). For comparison, very similar activation energies (0.19(2) and 0.30(3) eV) were reported by Adams and Rao,^28^ who also emphasized that hops involving the Li2 (and Li4) sites contribute significantly to the high ionic conductivity of LGPS at room temperature, effectively rendering it an isotropic ionic conductor.

Further studies on Li^+^ mobility in LGPS have identified local energy barriers that the ions must overcome both along the channels and within the *ab*-plane. While early investigations based on single-ion hopping models reported unrealistically high barriers of up to 0.47 eV along the *c*-axis,^9^ more recent studies have revealed significantly lower values.^3,12^ Recently, Huang et al.^12^ used neural network-assisted molecular dynamics simulations and calculated an activation energy of 0.083 eV for intrachannel Li^+^ diffusion, which supports the value of Yajima et al.^3^ found (0.09 eV) by the one-particle potential analysis. These values, as mentioned above, are in excellent agreement with those directly measured by ^7^Li SAE NMR in this work. More precisely, Yajima et al. report, as mentioned above, a very low activation energy of 0.03 eV for the strictly localized Li1–Li1 exchange.^3^ In contrast, the value of 0.09 eV corresponds to Li1–Li3 hopping, where the Li3 site, according to their neutron diffraction data, is a split site that undergoes further splitting (Li3a, Li3b) upon cooling to 10 K.^3^

Reported values for single-ion hopping along the Li1–Li3 channels range from as high as 0.47 eV to as low as 0.09 eV — discrepancies that do not align with experimental activation energies from conductivity measurements, typically around 0.3 eV. These inconsistencies sparked discussions about the rôle of strongly correlated ion motion in reconciling theory with experiment. Indeed, He et al. demonstrated that such correlations can lower the barrier from 0.47 to 0.2 eV,^9^ a trend reflected in the activation energies obtained by SAE NMR and in the stretched shape of the corresponding decay curves. Conversely, Yajima et al. showed that collective motions can also increase the barrier, namely from 0.09 to 0.33 eV.^3^ As emphasized by Yajima et al., who investigated the crystallographic structure of LGPS using neutron diffraction, precise structural information is essential for reliable and accurate predictions.^3^

While molecular dynamics simulations^27^ predict activation energies determining Li^+^ exchange within the *ab*-plane in good agreement with experimental values (see Figure 7), Yajima et al. report on activation barriers as high as 0.41 and 0.59 eV for correlated Li^+^ hopping involving the Li4 and the Li2 sites.^3^ Huang et al., based on neural network-assisted molecular dynamics simulations, identified for the Li2 and Li4 pathways activation barriers of 0.34 and 0.23 eV, see above. The latter value is close to the values seen by ^7^Li NMR (0.275 eV, 0.279 eV), as is shown in Figures 3b and 7. For comparison, Weber et al.^42^ calculated values as low as 0.12 and 0.13 eV for pathways involving the Li2 and Li4 sites. Overall, while at low temperatures the anisotropic nature of Li^+^ self-diffusion seems to be present, at sufficiently high temperatures, isotropic Li^+^ diffusion is expected for LGPS.

The high activation energy associated with exchange processes involving the Li2 site may be related to the final-state amplitudes observed in SAE NMR and the additional averaging seen in ^7^Li NMR quadrupolar intensities, as discussed above. The clear involvement of the Li2 site in Li^+^ diffusion at elevated temperatures is also supporting earlier results claiming 3D isotropic transport behavior in LGPS.^42^ The consecutive change in activation energy, and thus the transition from one-dimensional to three-dimensional ionic conduction, is illustrated in Figure 7, underscoring the strength of nucleus-specific NMR techniques in probing ion dynamics across a broad temperature range. These results enrich the dynamic understanding of LGPS and may encourage further computational studies to explore ion transport mechanisms at lower temperatures. Most notably, our study confirms the exceptionally low intrachannel Li^+^ activation barrier of 0.09 eV, as recently predicted by computational approaches.^3,12^

## Conclusions and Outlook

In this work, we employed ^7^Li SAE NMR to probe lithium-ion dynamics in LGPS over a wide temperature range, extending down to 90 K. The corresponding ^7^Li NMR line shapes have been recorded down to 7 K. This approach allowed us to directly observe extremely slow Li^+^ jump processes, on the order of one jump every three seconds, and to resolve distinct dynamic regimes characterized by activation energies of 0.09, 0.18, and 0.28 eV. The consecutive change in activation energies reflects a clear transition from spatially restricted 1D intrachannel diffusion to higher-dimensional transport involving Li1–Li3–Li4 exchange and the activation of Li2 sites.

The exceptionally low activation energy of 0.09 eV, detected by SAE NMR and supported by recent simulation studies, highlights the efficiency of intrachannel Li^+^ exchange within the rigid LGPS framework. The excellent agreement of our experimental jump rates as determined from ^7^Li and ^31^P NMR relaxation with those from PFG NMR, QENS, and simulation data consolidates our multiscale view of Li^+^ diffusion in LGPS. These results not only validate computational predictions but also underscore the critical rôle of nucleus-specific NMR techniques in mapping complex ion transport mechanisms.

By providing a detailed, temperature-resolved picture of Li^+^ dynamics, our findings lay the groundwork for future computational and experimental studies aimed at a deeper understanding of the elementary jump processes in LGPS-type electrolytes. To further improve transport properties, one could envision facilitating a smoother transfer of Li ions between channels and interchannel positions, for example via targeted doping, which might shift these transitions to lower temperatures and reduce the activation energy associated with 3D ion hopping in LGPS. Future experimental work could also explore the role of attempt frequencies and their connection to phonon modes in LGPS-type materials, providing insight into the changes in Arrhenius prefactors revealed in our study.

## Supplementary Material



## Notes

^7^Li and ^31^P NMR spin–lattice relaxation rates; peak positions; and activation energies (PDF)
