# Supplementary material for: Mapping the Various Li+ Jump Pathways in Li10GeP2S12: From Ultraslow Exchange to High-Temperature Diffusion
Source: J Am Chem Soc. 2025 Oct 14;147(42):38215–24. doi: 10.1021/jacs.5c10283 (PMC12550845; doi:10.1021/jacs.5c10283)
Supplement: Supplementary file 1 [file ja5c10283_si_001.pdf]

Supporting Information

Mapping the Various Li^+ Jump Pathways in $Li_{10}GeP_2S_{12}$: From Ultraslow Exchange to High-Temperature Diffusion

Annika Marko,[1] Katharina Hogrefe,[1] Lukas Schweiger,[1] Florian Stainer,[1] Jana Königsreiter,[1] Jonas Spychala,[1] Jakob Schwaiger,[1] Paul Heitjans,[2] Bernhard Gadermaier,[1] and H. Martin R. Wilkening[1]*

Graz University of Technology, Institute of Chemistry and Technology of Materials (NAWI Graz), Stremayrgasse 9, 8010 Graz, Austria

* corresponding authors: wilkening@tugraz.at

Crystal structure of LGPS. Figure S1 shows a magnified view of panels b, c, and d of Figure 1, providing better visibility of the possible Li diffusion pathways and atomic connectivities.

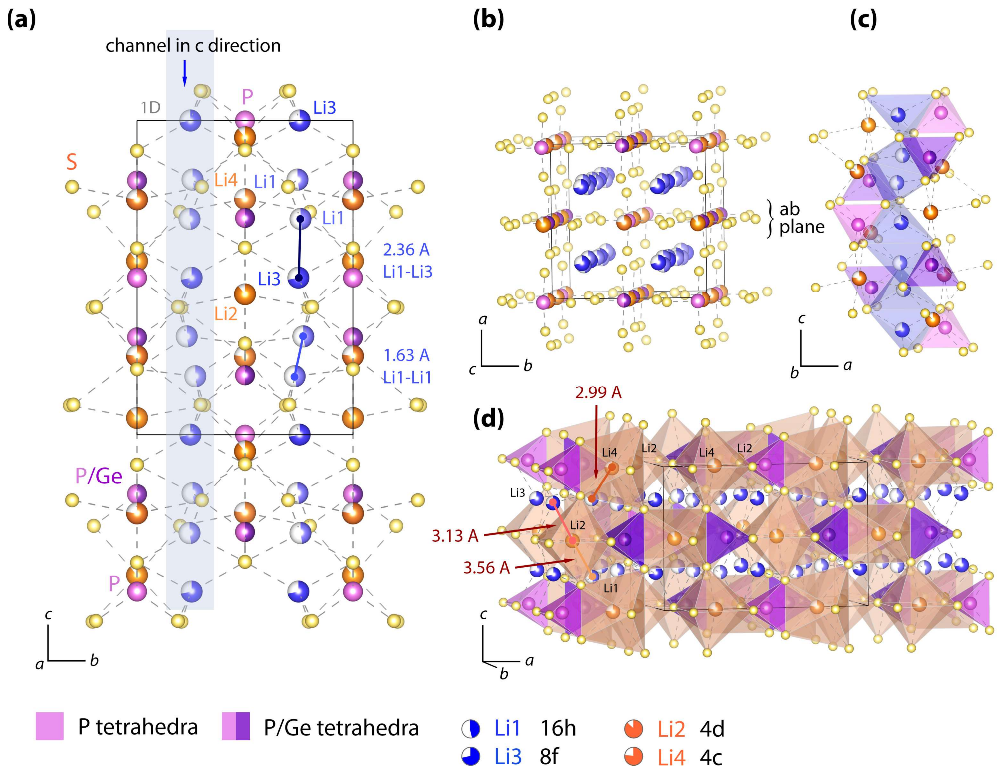

Figure S1: Crystal structure of $Li_{10}GeP_2S_{12}$ ($P4_2/nmc$). In (a) we highlight the channel-like arrangement of the Li1 and Li3 sites along the *c*-axis. The Li1-Li1 distance is 1.63 Å, while for Li1-Li3 the distance increased to 2.36 Å. (b) View along the *c*-axis to visualize the ab-plane with the Li2 and Li4 positions together with the P and Ge atoms in the same plane. (c) Li1 and Li3 tetrahedra are connected by sharing common edges. In (d) we highlighted the closest distances between Li1, Li4 and the Li positions in the *ab*-plane: Li1-Li4 (2.99 Å), Li3-Li2 (3.13 Å), Li1-Li2 (3.56 Å). Distances between Li3 and Li4 turn out to be rather long (> 4 Å). The dashed lines show the connections to sulfur anions and are just drawn to guide the eye.

Hopping along the Li1-Li3 channels is governed by activation energies of 0.09 and 0.18 eV, see main text. Jumps involving the non-channel sites (Li2 and Li4) have to overcome barriers as high as 0.28 eV, see main text.

NMR relaxation rates. The Li^+ jump rates shown in Figure 7, deduced from diffusion-induced NMR spin-lattice relaxation rates, were extracted from the rate peaks shown in Figure S2. For the ^{7}Li NMR rate peak $R_1(1/T)$, recorded at $\omega_0/2\pi = 116$ MHz) and for the ^{31}P NMR rate peak recorded in the rotating-frame of reference ($\omega_1/2\pi = 20$ kHz locking frequency, $\omega_0/2\pi = 121$ MHz), we observed distinct maxima.

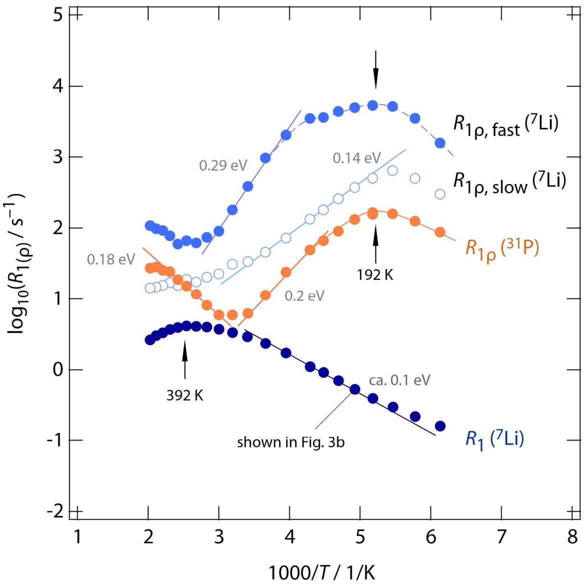

Figure S2: ^{7}Li (116 MHz) and ^{31}P (121 MHz) NMR spin-lattice relaxation rates of polycrystalline $Li_{10}GeP_2S_{12}$ recorded in both the laboratory frame and the rotating reference frame. Jump rates have been deduced for the distinct maxima seen in ^{7}Li (R_1) and ^{31}P ($R_{1\rho}$) diffusion-induced NMR spin-lattice relaxation in the laboratory frame and under spin-lock conditions, respectively. Experimental details are given in the main text. The jump rates determined have been included in Figure 7.

These maxima enabled us to reliably deduce the jump rates at $T = T_{max}$ where the peak appears on the temperature scale. At T_{max}, the motional correlation rate $1/\tau_c$, which is within a factor of two equal to the jump rate $1/\tau$, is given by $\tau \times \omega_0 \approx 0.62$ (for R_1) and $\tau \times \omega_1 \approx 0.5$ (for $R_{1\rho}$). A brief introduction into the basics of spin-lattice relaxation NMR is given elsewhere.[1-3]

In the case of ^{7}Li NMR $R_{1\rho}$ we observed magnetization transients $M_{xy}(t_{lock})$ that follow a two-step decay behavior leading to the rates shown in Figure S2. This behavior, presumably expected for spin-3/2 nuclei exposed to sufficiently fast diffusion,[4] makes it difficult to precisely determine T_{max} for the rather broad $R_{1fast}(1/T)$ rate peak, which, nonetheless, seems to appear at the same position as that of the corresponding ^{31}P NMR rates, as indicated by the vertical arrows. For the slower component, R_{1slow}, the peak $R_{1slow}(1/T)$ is shifted slightly toward lower temperatures. The corresponding jump rate would still support align with the Arrhenius line depicted in Figure 7.

Activation energies, as deduced from the high-temperature flanks of the various ^{7}Li and ^{31}P NMR rate peaks $R_{1(\rho)}(1/T)$ shown in Figure S2, can be broadly grouped into three categories: 0.10–0.14 eV, 0.20 eV, and 0.29 eV. The highest value (0.29 eV) corresponds to the high-temperature regime seen in Figure 7, whereas the other two groups roughly match the Arrhenius-like behavior observed at lower temperatures (see also Figure 7). The increasing $R_{1\rho}$ rates and the value of 0.18 eV from ^{31}P NMR at higher temperatures aligns with the behaviour also seen in

^{7}Li SAE NMR, see Figure 3b. It should be noted that, in contrast to other cases, spin-lattice relaxation in the present system is less effective at distinguishing between different diffusion pathways. This finding stands in contrast to other compounds exhibiting multiple motional processes, where clearer separation was possible.[5] As a result, the observed activation energy groups here indicate more gradual transitions between the processes.

Interestingly, ^{7}Li SAE NMR reveals the lowest activation energy (0.09 eV; see Figure 3b and Figure 7), as it allows non-diffusive background contributions, commonly affecting spin-lattice relaxation measurements, to be effectively separated. The difference between R_1 and $1/\tau_{SAE}$ at low temperatures is clearly illustrated in Figure 3b.

To convert the calculated and experimental diffusion coefficients D (from PFG NMR and QENS) into jump rates shown in Figure 7, we used the very-well known relationship between D and $1/\tau$: $D = a^2/(2d\tau)$. d is the dimensionality of the process (see Figure 7) and a represent the jump distance as is illustrated in Figure 1c. As an example, for the ('2D') diffusion process between (Li1, Li3) and Li4, a would be approximately 3 Å.

This conversion assumes a correlation factor of 1 between tracer and self-diffusion coefficients. While, in principle, such general factors in the form of adjustment parameters could be extracted from Figure 7, we consider this to be an overinterpretation of our data. Disregarding the minor deviations between rates obtained from the different methods (see Figure 7), one might argue that correlation effects play only a minor rôle in LGPS, as the results align well with the (simple) Einstein-Smoluchowski relation. However, this primarily applies at higher temperatures and does not account for Li^+ motion within the channels, where correlated dynamics are expected — a view also supported by the stretched shape of the SAE NMR decay functions and the agreement between activation energies from SAE NMR with those calculated, *e.g.*, by Huang *et al.*[6] In theoretical studies (see main text), such correlation or interrelation effects are explicitly considered when calculating activation energies for the intrachannel motional processes. Whether such effects manifest, in general, at higher or lower temperatures remains a matter of debate.

References

1. Gombotz, M.; Hiebl, C.; Stainer, F.; Wilkening, H. M. R., Solids with Two Mobile Ions: Proton H^+ Self-Diffusion in Li-H Exchanged Garnet-Type $Li_7LaZrTaO_{12}$ as Seen by Solid-State ^{1}H NMR Relaxation. *J. Phys. Chem. C* **2023**, *127*, 10960-10967.

2. Stainer, F.; Wilkening, H. M. R., Two-Dimensional Li^+ Ionic Hopping in Li_3InCl_6 as Revealed by Diffusion-Induced Nuclear Spin Relaxation. *Phys. Rev. B* **2024**, *109*.

3. Wilkening, M.; Heitjans, P., From Micro to Macro: Access to Long-Range Li^+ Diffusion Parameters in Solids via Microscopic ^{6}Li, ^{7}Li Spin-Alignment Echo NMR Spectroscopy. *Chem. Phys. Chem.* **2012**, *13*, 53-65.

4. Wimperis, S.; Rudman, G. E.; Johnston, K. E., Biexponential $I = 3/2$ Spin-Lattice Relaxation in the Solid State: Multiple-Quantum Li NMR as a Probe of Fast Ion Dynamics. *J. Phys. Chem. C* **2024**, *128*, 5453-5460.

5. Tapler, D.; Gadermaier, B.; Spychala, J.; Stainer, F.; Marko, A.; Königsreiter, J.; Hogrefe, K.; Heitjans, P.; Wilkening, H. M. R., Unraveling Ultrafast Li-Ion Dynamics in the Solid Electrolyte $LiTi_2(PS_4)_3$ by NMR down to Cryogenic Temperatures. *J. Am. Chem. Soc.* **2025**, *147*, 20023-20032.

6. Huang, Y.; Zhao, D.; Deng, M.; Shen, H., Revisiting the In-Plane and In-Channel Diffusion of Lithium Ions in a Solid-State Electrolyte at Room Temperature Through Neural Network-Assisted Molecular Dynamics Simulations. *Phys. Chem. Chem. Phys.* **2025**, *27*, 3243-3252.
